# Anti-Inflammatory Effects of Antarctic Lichen *Umbilicaria antarctica* Methanol Extract in Lipopolysaccharide-Stimulated RAW 264.7 Macrophage Cells and Zebrafish Model

**DOI:** 10.1155/2021/8812090

**Published:** 2021-02-16

**Authors:** Ju-Mi Hong, Jung Eun Kim, Seul Ki Min, Kyung Hee Kim, Se Jong Han, Joung Han Yim, Hyun Park, Jin-Hyoung Kim, Il-Chan Kim

**Affiliations:** ^1^Division of Life Sciences, Korea Polar Research Institute, Incheon 21990, Republic of Korea; ^2^Department of Pharmacy, Sungkyunkwan University, Suwon 16419, Republic of Korea; ^3^Department of Polar Sciences, University of Science and Technology, Incheon 21990, Republic of Korea; ^4^Division of Biotechnology, Korea University, Seoul 02841, Republic of Korea

## Abstract

*Umbilicaria antarctica* (UA) is a member of the family Umbilicariaceae. To the best of our knowledge, no studies on its anti-inflammatory effects have been reported yet. In the present study, we examined its ability to suppress inflammatory responses and the molecular mechanisms underlying these abilities using lipopolysaccharide- (LPS-) stimulated RAW 264.7 cells and a zebrafish model of inflammation. We investigated the effects of UA on the production of nitric oxide (NO) and prostaglandin E_2_ (PGE_2_) in LPS-stimulated RAW 264.7 cells. To explore the anti-inflammatory mechanisms of UA, we measured the mRNA and protein expression of proinflammatory mediators in LPS-stimulated RAW 264.7 cells using quantitative RT-PCR and western blot analyses, respectively. UA significantly inhibited the production of NO, PGE_2_, interleukin- (IL-) 6, and tumor necrosis factor- (TNF-) *α* in the LPS-stimulated RAW 264.7 cells. It also suppressed the mRNA and protein expression of inducible nitric oxide synthase (iNOS), cyclooxygenase-2 (COX-2), and nuclear factor- (NF-) *κ*B activation in LPS-stimulated RAW 264.7 cells and tail pin-cutting-induced zebrafish model. Collectively, these findings indicate that UA significantly inhibits LPS-stimulated inflammatory responses. These effects were considered to be strongly associated with the suppression of NF-*κ*B activation. Overall, our results demonstrate that UA extract exerts strong anti-inflammatory activities in *in vitro* and *in vivo* models and suggest that UA may be an effective novel therapeutic agent for the treatment of inflammatory diseases.

## 1. Introduction

Inflammation is a complex biological response of the immune system that can be induced in the tissue by various factors in response to harmful stimuli such as pathogens, damaged cells, or irritants and is characterized by redness, swelling, fever, pain, and impaired function at the tissue level [1, 2]. Lipopolysaccharide (LPS) is a potent activator of macrophages, and the activated macrophages produce a variety of proinflammatory molecules, including prostaglandin E_2_ (PGE_2_) and nitric oxide (NO). Among these molecules, inducible nitric oxide synthase (iNOS) and cyclooxygenase-2 (COX-2) are the proinflammatory enzymes necessary for inflammation, and they produce the proinflammatory mediators NO and PGE_2_, respectively [3]. Moreover, LPS stimulation also leads to the generation of inflammatory cytokines such as tumor necrosis factor-*α* (TNF-*α*) and interleukin- (IL-) 6 [4]. These molecules can serve as potential biomarkers of inflammatory diseases and help in prognosis and therapeutic decision-making [5].

The nuclear factor-*κ*B (NF-*κ*B) pathway is triggered by a variety of stimuli, including LPS, pathogen-associated molecular patterns (PAMPs), intercellular inflammatory cytokines, and many enzymes [6]. LPS binds toll-like receptor-4 (TLR-4) on the cell surface of macrophages and leads to NF-*κ*B activation through phosphorylation of inhibitor *κ*B (I*κ*B) by a large multisubunit kinase complex comprising inhibitor *κ*B kinase- (IKK-) 1*α* and 2*β*, which stimulates a cascade of intracellular signaling events [7]. Phosphorylation of I*κ*B results in its subsequent ubiquitination and degradation by the 26S proteasome, allowing the translocation of NF-*κ*B to the nucleus, thereby regulating the expression of various genes. This pathway is involved in the production of inflammatory cytokines and recruitment of inflammatory cells to contribute to the inflammatory response [8].

*Umbilicaria antarctica* (UA) belongs to the family Umbilicariaceae and is a widespread and abundant lichen in the Antarctic. It is often seen on sheltered north-facing slopes and boulders, sometimes predominantly in the fruticose lichen and moss cushion subformation [9]. Extracts of *Umbilicaria* sp. contain several bioactive compounds with antioxidant, antimicrobial, anticancer, and antithrombotic activities, along with inhibitory effects on melanogenesis [10–16]. However, no studies have been reported on the anti-inflammatory effects of *Umbilicaria* sp. in LPS-stimulated RAW 264.7 cells and zebrafish model. As a part of our ongoing search to identify anti-inflammatory compounds from extracts of lichens from the Antarctic, we studied the anti-inflammatory mechanism of UA by measuring its effects on the levels of cytokines, proinflammatory enzymes, and NF-*κ*B activation upon LPS stimulation in RAW 264.7 and zebrafish model.

## 2. Materials and Methods

### 2.1. Preparation of Lichen Material

UA was collected from the Barton Peninsula around King George Island (S62°14′06.15^″^, W58°46′22.68^″^), Antarctica, in January 2017. The nucleotide sequence of its mitochondrial small subunit rDNA (mtSSU) was analyzed using BLAST for sequence similarity with the NCBI GenBank database. For studies, the lichen samples were completely dried and incubated with methanol (1 g/10 mL) under a dark light of 25°C for two weeks. Methanol was evaporated using a speed vacuum concentrator. The methanol extract of UA was dissolved in dimethyl sulfoxide (DMSO, 20 mg/mL stock, stored at −20°C) and diluted to the indicated concentrations.

### 2.2. Cell Culture

RAW 264.7 cells (KCLB, Seoul, Korea) were grown to confluence in Dulbecco's modified Eagle's medium (DMEM, Sigma-Aldrich, St. Louis, MO, USA) supplemented with 10% heat-inactivated fetal bovine serum (FBS, Invitrogen, Burlington, ON, Canada) and 1% (*w*/*v*) antibiotic-antimycotic solution (Invitrogen, Grand Island, NY, USA) in a humidified atmosphere with 95% air and 5% CO_2_ at 37°C.

### 2.3. Cytotoxicity Assay

Cell cytotoxicity was determined by MTT (3-(4,5-dimethyl-2-thiazolyl)-2,5-diphenyl-2*H*-tetrazolium bromide, Amresco, Solon, OH, USA) assay. RAW 264.7 cells were seeded at a density of 1 × 10^5^ cells/mL in 96-well plates and incubated in the presence of various concentrations of UA extract for 24 h. Then, 5 *μ*L of a 5 mg/mL MTT solution was added to the cells and incubated for 4 h at 37°C. After treating with 100 *μ*L of fresh DMSO for 10 min, the signal was read at 570 nm using a microplate reader (Thermo Scientific Inc., San Diego, CA, USA). Relative cell viability was calculated by comparing the absorbances of the treated and untreated control groups. All experiments were performed in triplicates.

### 2.4. Estimation of Nitric Oxide Concentration

Nitrite levels in the culture supernatants were determined using the Griess reagent (1% sulfanilamide, 0.1% N-(1-naphthyl)-ethylenediamine dihydrochloride, and 5% phosphoric acid). Briefly, 5 × 10^5^ cells/mL were seeded onto 96-well plates and treated with the indicated concentrations of UA extract at 37°C for 1 h before stimulation with LPS (0.5 *μ*g/mL, Sigma-Aldrich, CA, USA) in a final volume of 200 *μ*L. After 24 h, 100 *μ*L of cell culture supernatant was mixed with the same volume of Griess reagent and incubated at room temperature for 5 min before measuring the absorbance at 540 nm and deducing the concentration of nitrite. Sodium nitrite was used to generate a standard curve. The assay was performed in triplicates.

### 2.5. Enzyme-Linked Immunosorbent Assay (ELISA)

Levels of TNF-*α*, IL-6, and PGE_2_ in the culture medium were determined by ELISA using paired antibodies as described previously [17]. In brief, RAW 264.7 cells were seeded at a density of 5 × 10^5^ cells/well in 96-well plates and treated with various concentrations of UA extract (0, 10, 20, 40, and 80 *μ*g/mL) for 1 h, followed by stimulation with 0.5 *μ*g/mL LPS for 24 h. The levels of TNF-*α*, IL-6, and PGE_2_ in the culture supernatant were determined according to the manufacturer's instructions using a commercially available DuoSet ELISA kit (R&D Systems, MN, USA) and a prostaglandin E_2_ (Cayman Chemical, Ann Arbor, MI) assay kit, respectively. Concentrations were determined from a standard curve and expressed as pg/mL.

### 2.6. Quantitative Real-Time Polymerase Chain Reaction Analysis

Total RNA extraction and RT-qPCR were carried out as described in a previous study [18]. Single-strand cDNA was synthesized using 1 *μ*g of total RNA using oligo (dT) primers and M-MLV reverse transcriptase. The primer pairs used for each gene are listed in Table 1. RT-qPCR was performed by real-time monitoring the increase in the amount of bound SYBR-Green using a Rotor-Gene 6500 RT-PCR system (Corbett Research, Sydney, Australia). The thermal cycling conditions were as follows: 60 min at 37°C, 15 min at 72°C for cDNA synthesis, denaturation at 95°C for 10 s, annealing at 60°C for 15 s, and elongation at 72°C for 60 s (30 cycles). *β*-Actin was used as the reference gene to normalize gene expression levels. Data were collected using the Rotor-Gene 6500 detection system. The cycle threshold (Ct) values were determined using automated threshold analysis, and primer quality was assessed using melting curve analysis. The *Δ*Ct values were calculated by subtracting the average Ct value of *β*-actin from the average Ct values of iNOS, COX-2, TNF-*α*, IL-1*β*, IL-6, and IL-10 of the untreated sample (Supplementary Table S1–S9). The standard deviation of the Ct values and variance of the *Δ*Ct values were calculated, which were used to determine the *ΔΔ*Ct values. All experiments were performed in triplicates.

### 2.7. Immunoblot Analysis

Total cellular extracts were prepared according to our previous report [17], separated by SDS-PAGE on an 8% gel, and the protein bands were transferred to a polyvinylidene fluoride (PVDF, Millipore Corporation, Billerica, MA) membrane. The membrane was incubated with a blocking solution (5% skim milk) and then incubated with specific antibodies overnight at 4°C. These included anti-iNOS (1 : 1000 dilution, Enzo Life Sciences, NY, USA), anti-COX-2 (1 : 1000 dilution, Santa Cruz Biotechnology, CA, USA), anti-NF-*κ*B p65 (1 : 1000 dilution, Cell Signaling, CA, USA), phospho-I*κ*B-*α* (1 : 1000 dilution, Cell Signaling), anti-*β*-actin (1 : 1000 dilution, Cell Signaling), anti-PCNA (1 : 1000 dilution, Santa Cruz Biotechnology), and anti-GAPDH (1 : 1000 dilution, Santa Cruz Biotechnology). Membranes were washed three times with tris-buffered saline containing 0.1% Tween 20 (TBST) and then incubated with a 1 : 2000 dilution of horseradish peroxidase- (HRP-) conjugated secondary antibody (Santa Cruz Biotechnology) for 1 h at 25°C. Membranes were washed three times with TBST and then developed using an enhanced chemiluminescence (ECL) kit (Translab, South Korea). For quantitative analysis, densitometric values of different bands were determined using a LAS-3000 imager (Fujifilm, Japan).

### 2.8. Toxicity Studies Using Zebrafish Embryos

Normal zebrafish larvae in the epiboly stage were examined under a stereo microscope (SteREO Discovery V8, ZEISS, Jena, Germany). For treatment with each concentration, 10 eggs were selected in 3 replicates and individually transferred to 12-well plates with each well containing 2 mL of the test solution. The plates were incubated at 28°C with a 12 h light/dark cycle. The phenotypes of the embryos were recorded, and the survival rate, hatching rate, length of the body, and malformations were noted using a stereo microscope at 24, 48, 72, 96, and 120 h postfertilization (hpf). The survival and hatching rates were calculated as the proportion of successful survival and hatching in each replicate. The body length of zebrafish larvae was measured from the front of the head to the tip of the tail along the body axis using the iWORKs 2.0 series program. The heart rate was measured by counting the beats for 20 seconds under a stereo microscope.

### 2.9. Quantitative Real-Time Polymerase Chain Reaction Analysis in Zebrafish

Zebrafish larvae (120 hpf) were transferred to 12-well plates containing embryo medium with 10 individuals in each well. They were divided into normal control, model group, positive control, and UA treatment (0, 25, 50, and 100 *μ*g/mL) groups, with four wells per group. Tails of the larvae were cut off after anesthetizing them with 0.16% ethyl 3-aminobenzoate (tricaine, Sigma-Aldrich, CA, USA), except those from the normal control group. This was followed by treatment with UA extract (0, 25, 50, and 100 *μ*g/mL) for 24 h in a 28°C incubator. After washing the animals in DW three times, the total RNA from each zebrafish was isolated using TRIzol reagent (Molecular Research Center, Inc., OH, USA). For RT-PCR, 1 *μ*g total RNA was reverse transcribed into single-strand cDNA using oligo (dT) primers and M-MLV reverse transcriptase. The primers used in PCR are listed in Table 2. The relative levels of expression of target genes were quantified using housekeeping gene *β*-actin. All experiments were performed in triplicates.

### 2.10. Production of Reactive Oxygen Species (ROS) in Zebrafish Larvae due to Exposure to UA

Approximately 3 days postfertilization (dpf), larvae were transferred individually to the 12-well plate and exposed to different concentrations (0, 25, 50, and 100 *μ*g/mL) of UA extract followed by LPS (10 *μ*g/mL) treatment for 24 h. Generation of ROS was detected using 2′,7′-dichlorofluorescin diacetate (DCF-DA, Sigma-Aldrich) green fluorescent reagent. These live larvae were washed in embryo medium, incubated with DCF-DA (20 *μ*g/mL) solution for 1 h in dark at 28°C, again washed three times in embryo medium, and anesthetized with 0.16% tricaine to study using a fluorescent microscope (ZEISS, Jena, Germany). The fluorescence intensity of individual larva was quantified with the ImageJ program and calculated as compared to controls.

### 2.11. Statistical Analysis

Values are expressed as the mean ± standard error of the mean (SEM). The significance of the difference from the respective controls for each experimental test condition was assayed using Student's *t*-test for each paired experiment. The difference was considered statistically significant at ^∗^*P* < 0.05 and ^∗∗^*P* < 0.01.

## 3. Results

### 3.1. Effect of UA on Cell Viability

**Figure 1:**
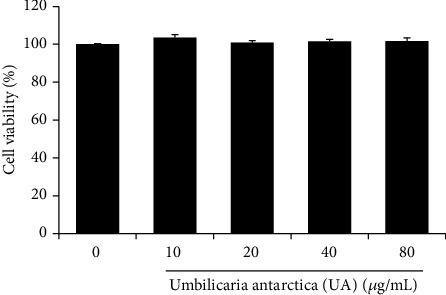
Effect of the methanolic extract of *U. antarctica* (UA) on the proliferation of RAW 264.7 cells. Cells were treated with various concentrations of UA extract for 24 h, and cell viability was measured by MTT assay. Three independent experiments were performed, and the data are presented as the mean ± SEM.

A precondition to study the biological activity of UA extract was to ensure that it did not exert harmful effects on cellular metabolism. To determine whether the UA extract affected cell viability, RAW 264.7 cells were incubated with 0, 10, 20, 40, and 80 *μ*g/mL extract for 24 h. No cytotoxic effects were observed during the experimental period in these cells at concentrations up to 80 *μ*g/mL of the extract (Figure 1). Therefore, we used up to 80 *μ*g/mL UA extract for subsequent experiments.

### 3.2. Reduction in NO Production by UA through Suppression of iNOS Expression in LPS-Stimulated RAW 264.7 Cells

**Figure 2:**
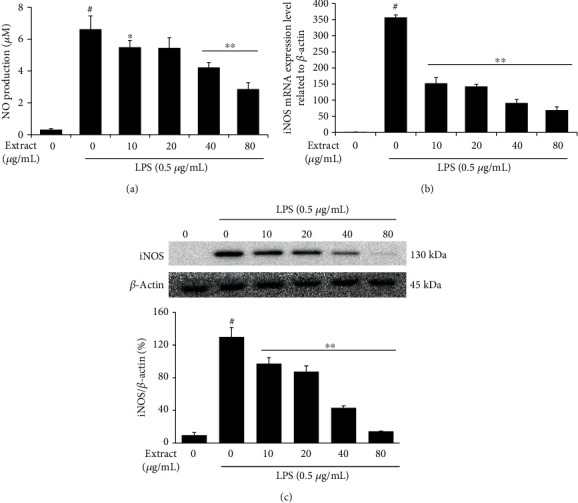
Inhibitory effects of UA extract on nitric oxide (NO) production and inducible-nitric oxide synthase (iNOS) expression in LPS-stimulated RAW 264.7 cells. (a) Cells were pretreated with the indicated concentrations of UA extract for 1 h prior to incubation with LPS (0.5 *μ*g/mL) for 24 h. The nitrite levels in the culture medium were measured by the Griess reaction. Three independent experiments were performed, and the data are presented as the mean ± SEM. ^∗^*P* < 0.05 and ^∗∗^*P* < 0.01 compared to the cells treated with LPS alone. (b) Cells were pretreated with UA extract for 1 h and then stimulated with LPS for 6 h. *β*-Actin expression was used as an internal control for quantitative RT-PCR. (c) Cells were pretreated with UA extract for 1 h and then induced with LPS for 24 h. Ten cell lysates were prepared, and the iNOS protein levels were analyzed on western blots. Expression of *β*-actin was used as an internal control for western blot analysis.

LPS-stimulated RAW 264.7 cells are known to increase NO production through iNOS activation induced by L-arginine [19]. To investigate the suppressive effects of UA extract on LPS-stimulated iNOS expression, cell lysates were analyzed for protein and mRNA expression using western blots and quantitative RT-PCR, respectively. Pretreatment with UA extract significantly suppressed the LPS-stimulated expression of iNOS mRNA and protein in a dose-dependent manner (Figures 2(b) and 2(c)). To measure the effect of UA extract on NO production, we estimated the amount of nitrite in the medium using the Griess reaction. As shown in Figure 2(a), the nitrite concentration in RAW 264.7 cells increased substantially upon LPS stimulation, while pretreatment with UA extract inhibited this in a dose-dependent manner. These results suggested that UA-mediated inhibition of NO production in the LPS-stimulated RAW 264.7 cells was associated with inhibition of iNOS expression at the transcriptional level.

### 3.3. Decline in PGE_2_ Production by UA Extract through Suppression of COX-2 Expression in LPS-Stimulated RAW 264.7 Cells

**Figure 3:**
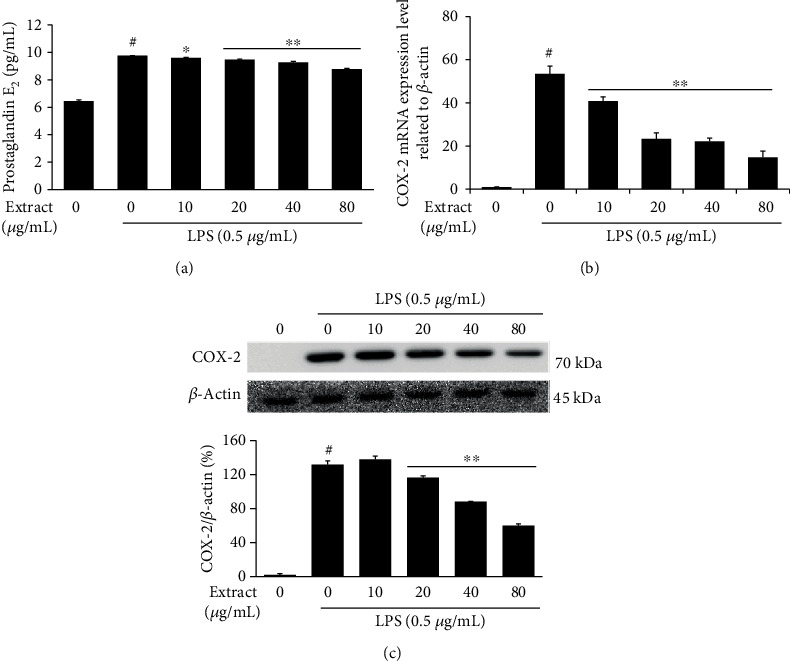
Inhibitory effects of UA extract on prostaglandin E_2_ (PGE_2_) production and cyclooxygenase-2 (COX-2) expression in LPS-stimulated RAW 264.7 cells. (a) Cells were treated with the indicated concentrations of UA extract for 1 h prior to incubation with LPS (0.5 *μ*g/mL) for 24 h. The PGE_2_ levels in the culture medium were measured using enzyme-linked immunosorbent assay (ELISA). Three independent experiments were performed, and the data are presented as the mean ± SEM. ^∗^*P* < 0.05 and ^∗∗^*P* < 0.01 compared to the cells treated with LPS alone. (b) Cells were treated with UA extract for 1 h and then induced with LPS for 6 h. *β*-Actin expression was used as an internal control for quantitative RT-PCR analysis. (c) Cells were treated with UA extract for 1 h and then induced with LPS for 24 h. Ten cell lysates were prepared, and the COX-2 protein expression levels were analyzed by western blotting. *β*-Actin expression was used as an internal control for western blots.

To determine whether UA extract could affect COX-2 expression in LPS-stimulated RAW 264.7 cells, we treated these cells with the extract, exposed them to LPS, and evaluated the expression levels of COX-2 protein and mRNA by western blot and quantitative RT-PCR, respectively. Results show that LPS-stimulated RAW 264.7 cells overexpressed COX-2 mRNA, and this was reduced when the cells were pretreated with UA extract (Figure 3(b)). Similarly, the expression of COX-2 protein was also suppressed by UA extract in these cells (Figure 3(c)).

PGE_2_ is a downstream product of COX-2, synthesized through the cyclooxygenase and prostaglandin synthase pathways [20]. We also evaluated the effect of UA extract on PGE_2_ production. Following the inhibition of COX-2 expression, UA extract decreased PGE_2_ production in LPS-stimulated RAW 264.7 cells in a dose-dependently manner (Figure 3(a)).

### 3.4. Effect of UA on the Production of Proinflammatory Cytokine in LPS-Stimulated RAW 264.7 Cells

**Figure 4:**
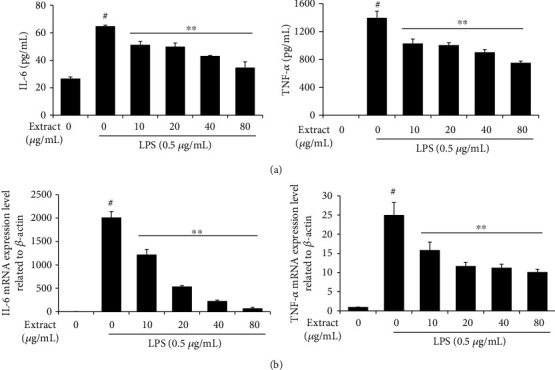
Effects of UA extract on the transcription and translation of interleukin- (IL-) 6 and TNF-*α* in LPS-induced RAW 264.7 cells. (a) Cells were treated with the indicated concentrations of UA extract for 1 h prior to incubation with LPS (0.5 *μ*g/mL) for 24 h. The level of IL-6 and TNF-*α* in the supernatant was determined by enzyme-linked immunosorbent assay (ELISA). (b) Cells were treated with UA extract for 1 h and then induced with LPS for 6 h. *β*-Actin expression was used as an internal control for quantitative RT-PCR analysis. Three independent experiments were performed, and the data are presented as the mean ± SEM. ^∗^*P* < 0.05 and ^∗∗^*P* < 0.01 compared to the cells treated with LPS alone.

To further examine the inhibitory effects of UA extract on LPS-stimulated production of proinflammatory cytokines, the protein and mRNA levels of IL-6 and TNF-*α* in LPS-treated RAW 264.7 cells, with and without pretreatment with UA extract, were measured by ELISA and quantitative RT-PCR, respectively. IL-6 and TNF-*α* were expressed in very low levels in normal control RAW 264.7 cells but increased significantly upon exposure to LPS. However, pretreatment with UA extract (0, 10, 20, 40, and 80 *μ*g/mL) significantly inhibited this upregulation in a dose-dependent manner (Figure 4(a)). With 80 *μ*g/mL UA extract, IL-6 and TNF-*α* production was decreased to less than half of that seen in the LPS-treated RAW 264.7 cells. Inhibitory effects of UA extract on the mRNA expression followed a similar pattern (Figure 4(b)). The mRNA levels of IL-6 and TNF-*α* were significantly increased by LPS, but pretreatment with UA extract markedly and dose-dependently decreased these. Thus, the UA extract inhibited both transcription and translation of proinflammatory cytokines in LPS-stimulated RAW 264.7 cells.

### 3.5. Effect of UA on p-I*κ*B and NF-*κ*B Activation in LPS-Stimulated RAW 264.7 Cells

**Figure 5:**
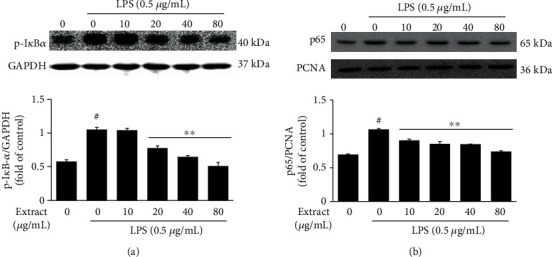
Effect of UA extract on the regulation of the nuclear factor- (NF-) *κ*B pathway. Cells were treated with the indicated concentrations of UA extract for 1 h, followed by induction with LPS (0.5 *μ*g/mL) for 30 min. Equal amounts of protein were analyzed using specific antibodies against p-I*κ*B and p65. Levels of p-I*κ*B and p65 protein in (a) cytosolic and (b) nuclear fractions measured by western blotting. Three independent experiments were performed, and the data are presented as the mean ± SEM. ^∗^*P* < 0.05 and ^∗∗^*P* < 0.01 compared to the cells treated with LPS alone. Expressions of GAPDH and PCNA were used as internal controls for western blot analysis.

Previous studies have suggested that NF-*κ*B is an important transcription factor that regulates iNOS, COX-2, and expression of inflammatory cytokines such as TNF-*α* and IL-6 [21]. We examined the effect of UA extract on LPS-stimulated NF-*κ*B activation. As shown in Figure 5, exposure to LPS triggered phosphorylation of I*κ*B-*α* and consequent nuclear translocation of NF-*κ*B p65. However, pretreatment with UA extract significantly attenuated the cytosolic level of phosphorylated I*κ*B-*α* (p-I*κ*B-*α*) and nuclear NF-*κ*B p65, compared to that in LPS-stimulated RAW 264.7 cells, in a dose-dependent manner. These results indicated that the UA extract exerts its inhibitory effect on the upregulation of NO, PGE_2_, iNOS, COX-2, TNF-*α*, and IL-6 levels by LPS, through inhibition of I*κ*B-*α* phosphorylation, resulting in the prevention of nuclear translocation of NF-*κ*B p65.

### 3.6. Effects of UA on Zebrafish Development

**Figure 6:**
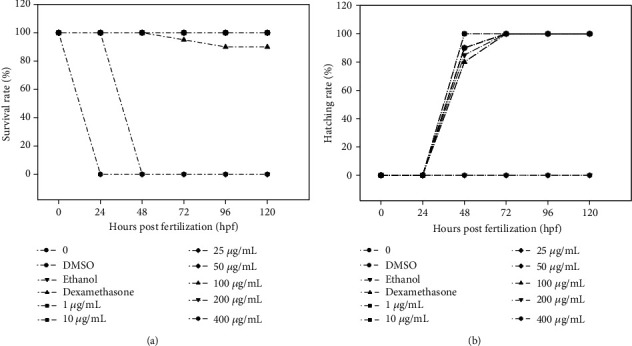
Effect of UA extract on development in zebrafish embryos for 120 hpf. Survival and hatching rates were monitored in zebrafish embryos after exposure at epiboly stage to various concentrations (1, 10, 25, 50, 100, 200, and 400 *μ*g/mL) of UA extract for 5 days. Experiments were performed in three replicates for each concentration.

**Figure 7:**
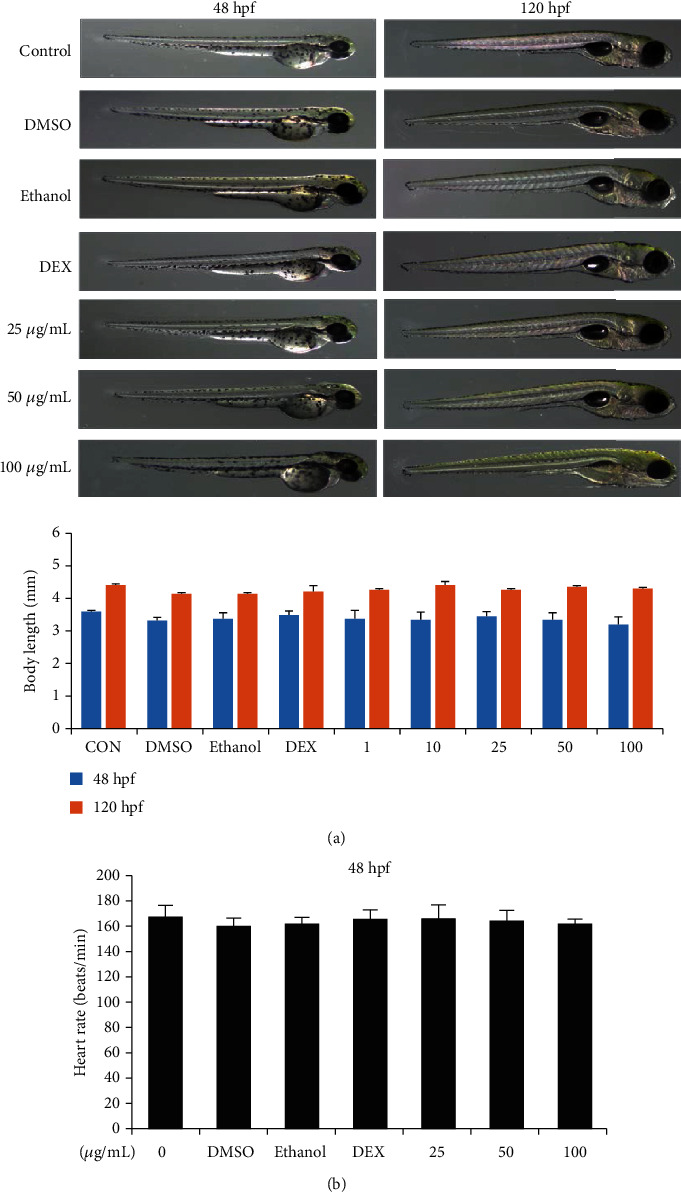
Effects of UA extract on embryonic development of zebrafish. (a) After treatment with various concentrations (0, 25, 50, and 100 *μ*g/mL) of UA extract, the phenotype and body length of zebrafish larvae in 48 hpf and 120 hpf were studied. (b) The effect of UA extract on the heartbeat rate at 48 hpf of zebrafish embryos.

To find out whether UA extract causes developmental toxicity in zebrafish embryos, mortality rates, malformations, and morphological abnormalities were studied in the treated embryos at 24, 48, 72, 96, and 120 hpf, and their survival was determined by the presence of a heartbeat. Our results showed that exposure to UA extract at a concentration of 100 *μ*g/mL at 120 hpf did not affect the survival rates significantly (Figure 6(a)). However, survival decreased dramatically to almost 0% when treated at 24 and 48 hpf, in the 200 and 400 *μ*g/mL groups, respectively. Because of this, subsequent sets of experiments did not involve treatment with 200 and 400 *μ*g/mL UA extract, and only the concentrations below 100 *μ*g/mL were used. These embryos hatched normally, at a high rate (Figure 6(b)). Furthermore, the morphology and heart rate after exposure to UA extract for 0-120 h were not significantly different from the normal controls (Figure 7).

### 3.7. Inhibitory Effect of UA on Inflammatory Factors in Zebrafish

**Figure 8:**
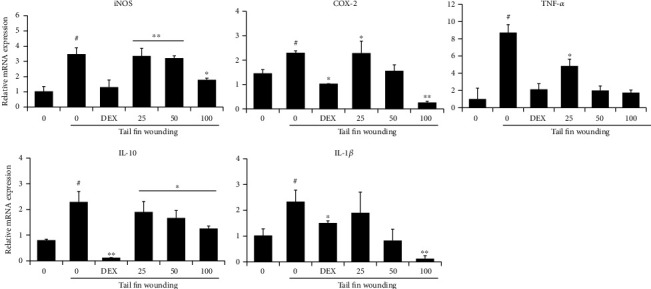
Effect of UA extract on inflammatory gene expression in zebrafish larvae. Tail fin from 3 dpf zebrafish larvae was wounded, and mRNA levels of iNOS, COX-2, TNF-*α*, IL-10, and IL-1*β* were determined by quantitative real-time PCR. Gene expression was normalized against that of *β*-actin. Data represent the average of the three replicates, and *P* values were calculated using *t*-test. ^∗^*P* < 0.05 and ^∗∗^*P* < 0.01.

To confirm the anti-inflammatory effect of UA extract, we analyzed the expression levels of several immune response genes, including proinflammatory genes such as iNOS, COX-2, TNF-*α*, IL-10, and IL-1*β*. Inflammatory response was elicited by injuring the zebrafish tail through amputation. Our results demonstrated that the expression of proinflammatory genes increased significantly after injury, compared to uncut control larvae (Figure 8). When they were exposed to UA extract for 24 h after tail cutting, mRNA levels of iNOS, COX-2, TNF-*α*, IL-10, and IL-1*β* were suppressed in a dose-dependent manner. In particular, COX-2 and TNF-*α* expression were inhibited to a greater extent, compared to dexamethasone-treated larvae, exposed to 100 *μ*g/mL UA extracts.

### 3.8. Production of Reactive Oxygen Species (ROS) in Zebrafish Larvae after UA Exposure

**Figure 9:**
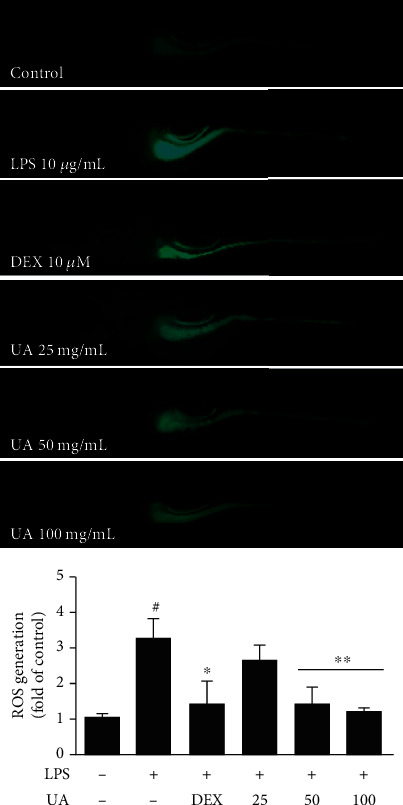
Effect of UA extract on LPS-induced ROS generation in zebrafish larvae. (a) Zebrafish larvae were treated with 25, 50, and 100 *μ*g/mL UA extract and 10 *μ*g/mL LPS for 24 h. The levels of ROS were observed under a fluorescence microscope after staining with DCF-DA. (b) The fluorescence intensities in individual zebrafish larvae were quantified. Data represent the average of the three replicates, and *P* values were calculated using *t*-test. ^∗^*P* < 0.05 and ^∗∗^*P* < 0.01.

Recent studies have reported that zebrafish were used for simple and quick evaluation of anti-inflammatory activity against LPS-stimulated inflammation *in vivo* [22]. To detect LPS-stimulated ROS generation in zebrafish larvae, an oxidation-sensitive fluorescent dye, DCF-DA, was used.  Figure 9 shows that zebrafish exposed to LPS had significantly higher ROS than the control group. However, when zebrafish were treated with LPS and UA extract, ROS generation decreased in a dose-dependent manner.

## 4. Discussion

In the present study, we evaluated the anti-inflammatory effect of a natural product extracted from the lichen UA on the inflammation-related mechanisms in LPS-stimulated RAW 264.7 cells. To understand the related molecular mechanisms, we investigated how the production of NO, PGE_2_, IL-6, and TNF-*α*, the expression of iNOS and COX-2, and the activation of NF-*κ*B pathway proteins are affected by exposure to UA extract.

NO gas is a soluble free radical with several physiological and pathophysiological effects [23]. It is generated from L-arginine by the enzymes of the nitric oxide synthase (NOS) family, comprising endothelial NOS (eNOS), neuronal NOS (nNOS), and inducible NOS (iNOS) [24]. iNOS is a calcium-independent enzyme that produces higher amounts of NO, unlike eNOS and nNOS, which are calcium-dependent enzymes, responsible for the production of low, physiological amounts of NO [23]. Although it plays an important role in defending against various pathogens, NO produced by iNOS under inflammatory conditions is harmful and causes tissue damage, autoimmune diseases, and inflammation-related diseases [25]. Therefore, inhibition of iNOS expression in response to inflammatory conditions may be a useful therapeutic strategy for reducing inflammation. Our results indicate that treatment with UA extract decreased the iNOS levels significantly, in a dose-dependent manner at both mRNA and protein levels, and also reduced NO production (Figure 2).

Prostaglandins are involved in physiological or pathological changes in the body, such as regulation of immune responses, blood pressure, platelet aggregation, and neurotransmitter release [26]. PGE_2_ is one of the most studied prostaglandins and is involved in the processes leading to typical inflammatory symptoms, like redness, pain, swelling, and fever [27]. Synthesis of PGE_2_ begins with the release of arachidonic acid from the membrane phospholipids by the enzymatic action of phospholipase A2. Following its release, arachidonic acid is converted to prostaglandin G_2_ (PGG_2_), which in turn is converted to an unstable metabolite prostaglandin H_2_ (PGH_2_) by cyclooxygenase (COX) enzymes [28]. COX consists of two isoenzymes: COX-1, which is responsible for housekeeping functions such as protection of gastric epithelial cells and homeostasis, and COX-2, induced by inflammatory stimuli, hormones, and growth factors, is an important player in inflammation and inflammatory diseases like cancer [29]. We examined the effects of UA extract on the expression of PGE_2_ and COX-2 in the inflammatory response. Our results demonstrated that UA extract inhibited PGE_2_ production by reducing the expression of COX-2 in LPS-stimulated RAW 264.7 cells (Figure 3).

Our results also showed that UA extract suppressed the production and expression of the proinflammatory cytokines IL-6 and TNF-*α*, in a dose-dependent manner (Figure 4). These cytokines induce inflammation through macrophages. IL-6 is strongly activated in the acute phase response that contributes to both systemic and local inflammatory responses [30]. TNF-*α* also plays a significant role in the immune system during inflammation, differentiation, cell proliferation, and apoptosis [31]. Therefore, inhibition of production or function of these cytokines can be regarded as a key mechanism of inflammation suppression in RAW 264.7 cells.

NF-*κ*B is a transcription factor important in regulating the expression of inflammatory mediators including iNOS, COX-2, IL-6, and TNF-*α* in inflammatory processes [32]. NF-*κ*B signaling is activated by inducing I*κ*B-*α* phosphorylation and degradation by the IKK complex, upon stimulation with molecules such as LPS from bacterial pathogens. NF-*κ*B then translocates to the nucleus and binds to its cognate sequence in the promoter region of many inflammatory genes, including those encoding iNOS, COX-2, IL-6, and TNF-*α*, to promote transcription [33]. To understand the mechanism by which UA extract affects cytokines and exerts its anti-inflammatory effects, we evaluated the phosphorylation status of I*κ*B-*α* and the level of nuclear NF-*κ*B p65 in the LPS-stimulated RAW 264.7 cells. Our results indicate that UA inhibits I*κ*B-*α* degradation and the consequent nuclear translocation of NF-*κ*B p65 in LPS-stimulated RAW 264.7 cells in a dose-dependent manner (Figure 5).

Zebrafish is a popular *in vivo* model system to evaluate drug efficacy, toxicity, and stability. We used it to confirm the anti-inflammatory effects of UA extracts. An important advantage of the zebrafish model is that its transparent body allows physical examination of internal organs in live specimen. In addition, it shows similar physiological similarities to mammals, and large populations can be used for experiments due to their small size, low cost, high reproduction rate, rapid growth, and requirement of smaller amounts of test compounds, as compared to other model animals such as mice and rats [34, 35]. In this study, we found that cutting the tail fin increased the expression of inflammatory mediators including iNOS, COX-2, TNF-*α*, IL-10, and IL-1*β* in zebrafish. We also found that UA extracts inhibited the expression of inflammatory mediators in a dose-dependent manner (Figure 8). To our knowledge, this is the first report to prove the anti-inflammatory effects of UA extracts in the zebrafish model.

In traditional medicine, a whole plant extract or a combination of plant extracts is often used instead of a single compound. Several studies have shown that disease resistance is less likely to occur if a combination of complex compounds is used instead of a single active ingredient [36–38]. Therefore, several plant extracts are often used together for medical purposes as they possess a more complex composition than single plant extracts. However, it is important to assess the interactions between the compounds, as well as the effectiveness of the crude extracts [39, 40]. In our study, a crude extract from UA was used to investigate the potential anti-inflammatory effect of the lichen. Recent studies have assessed the anti-inflammatory efficacy of polysaccharides isolated from *Umbilicaria esculenta* by significantly inhibiting concentration-dependent NO production in RAW 264.7 cells [41, 42]. In addition, several studies have demonstrated that bioactive compounds isolated from lichen have antioxidant and antitumor properties [43, 44]. Therefore, further studies are required to demonstrate the underlying mechanism of immune activity through positive interactions known as potentiation or synergistic effects of new components of *U. antarctica* extract.

## 5. Conclusion

In conclusion, the results of the present study demonstrated that the crude UA extract had an anti-inflammatory effect *in vitro*. It downregulated inflammatory mediators in LPS-stimulated RAW 264.7 cells by attenuating NF-*κ*B activation, thereby modulating the NF-*κ*B pathway. Therefore, our results suggested that UA extract could be an effective therapeutic agent for the treatment of inflammatory diseases such as rheumatoid arthritis, asthma, and cancer.

## Figures and Tables

**Table 1:** Primer used to analyzed inflammatory gene expression in RAW 264.7.

Gene	Primer sequence (forward 5′→3′)	Primer sequence (reverse 5′→3′)
iNOS	5′-GGA GCC TTT AGA CCT CAA CAG A-3′	5′-TGA ACG AGG AGG GTG GTG-3′
COX-2	5′-GAA GTC TTT GGT CTG GTG CGT G-3′	5′-GTC TGC TGG TTT GGA ATA GTT GC-3′
IL-6	5′-GAG GAT ACC ACT CCC AAC AGA CC-3′	5′-AAG TGC ATC GTT GTT CAT ACA-3′
TNF-*α*	5′-CAT CTT CTC AAA ATT CGA GTG ACA A-3′	5′-TGG GAG TAG ACA AGG TAC AAC CC-3′
*β*-Actin	5′-TGT TTG AGA CCT TCA ACA CC-3′	5′-AGT CTG TCA GGT CCC GGC C-3′

**Table 2:** Primer used to analyzed inflammatory gene expression in zebrafish.

Gene	Primer sequence (forward 5′→3′)	Primer sequence (reverse 5′→3′)
iNOS	5′-CTG CGG TGG AAT GAA CAT GG-3′	5′-TCT CCA GCT TCT ACC TCG CTC-3′
COX-2	5′-TGC TGC TTT GGT GGA CTT AC-3′	5′-TTC AGA GGA GGG CTA TTG TCA G-3′
TNF-*α*	5′-TCT CAG GGC AAG AAA TTC GAC-3′	5′-TCT CAC TGC ATC GGC TTT GT-3′
IL-10	5′-TAG GAT GTT GCT GGG TTG GAC-3′	5′-TAG TGT GAT GGA TGG ACG GG-3′
IL-1*β*	5′-GCA CGG CTA TTC AGA GAT GGT-3′	5′-CCA AGA ATA AGC AGC ACT TGG G-3′
*β*-Actin	5′-GCC ACC TTA AAT GGC CTA GCA-3′	5′-GCC ATA CAG AGC AGA AGC CA-3′

## Data Availability

The datasets used and/or analyzed during the current study are available from the corresponding author on reasonable requests.

## Supplementary Materials

Supplementary Materials The supplementary material file contains the following: (1) Table S1: results of the iNOS expression in LPS-stimulated RAW 264.7 cells. (2) Table S2: results of the COX-2 expression in LPS-stimulated RAW 264.7 cells. (3) Table S3: results of the IL-6 expression in LPS-stimulated RAW 264.7 cells. (4) Table S4: results of the TNF-*α* expression in LPS-stimulated RAW 264.7 cells. (5) Table S5: results of the iNOS expression in zebrafish larvae. (6) Table S6: results of the COX-2 expression in zebrafish larvae. (7) Table S7: results of the TNF-*α* expression in zebrafish larvae. (8) Table S8: results of the IL-10 expression in zebrafish larvae. (9) Table S9: results of the IL-1*β* expression in zebrafish larvae.
