# Supplementary material for: Anti-Inflammatory Effects of Antarctic Lichen Umbilicaria antarctica Methanol Extract in Lipopolysaccharide-Stimulated RAW 264.7 Macrophage Cells and Zebrafish Model
Source: Biomed Res Int. 2021 Feb 16;2021:8812090. doi: 10.1155/2021/8812090 (PMC7902135; doi:10.1155/2021/8812090)
Supplement: Supplementary Materials — The supplementary material file contains the following: (1) Table S1: results of the iNOS expression in LPS-stimulated RAW 264.7 cells. (2) Table S2: results of the COX-2 expression in LPS-stimulated RAW 264.7 cells. (3) Table S3: results of the IL-6 expression in LPS-stimulated RAW 264.7 cells. (4) Table S4: results of the TNF-α expression in LPS-stimulated RAW 264.7 cells. (5) Table S5: results of the iNOS expression in zebrafish larvae. (6) Table S6: results of the COX-2 expression in zebrafish larvae. (7) Table S7: results of the TNF-α expression in zebrafish larvae. (8) Table S8: results of the IL-10 expression in zebrafish larvae. (9) Table S9: results of the IL-1β expression in zebrafish larvae. [file 8812090.f1.docx]

## Supplementary Materials

**Table S1: Results of the iNOS expression in LPS-stimulated RAW 264.7 cells.**

| **sample** | **β-actin** | **average** | **β-actin Std.** | **iNOS** | **iNOS/β-actin** | **average (ΔCt)** | **ΔΔCt** | **2^-(ΔΔCt)** | **ΔΔCt average** | **ΔΔCt STD** | **t-test** | **p value** |
| --- | --- | --- | --- | --- | --- | --- | --- | --- | --- | --- | --- | --- |
| - (control) | 23.42 | 23.4766667 | 0.0981 | 29.58 | 6.103333333 | 6.0233333 | 0.08 | 0.946057647 | 1.0055258 | 0.132 |  |  |
|  | 23.59 |  |  | 29.29 | 5.813333333 |  | -0.21 | 1.156688184 |  |  |  |  |
|  | 23.42 |  |  | 29.63 | 6.153333333 |  | 0.13 | 0.91383145 |  |  |  |  |
| + (LPS) | 23.36 | 23.4833333 | 0.2136 | 21.06 | -2.423333333 | -2.456667 | -8.44666667 | 348.8992453 | 357.10763 | 7.537 | 0.000000 | # |
|  | 23.36 |  |  | 21.02 | -2.463333333 |  | -8.48666667 | 358.7081382 |  |  |  |  |
|  | 23.73 |  |  | 21 | -2.483333333 |  | -8.50666667 | 363.7155172 |  |  |  |  |
| 10 (+LPS) | 23.48 | 23.6666667 | 0.265 | 22.27 | -1.396666667 | -1.22 | -7.42 | 171.254727 | 152.19334 | 17.71 | 0.000025 | ** |
|  | 23.97 |  |  | 22.47 | -1.196666667 |  | -7.22 | 149.0858991 |  |  |  |  |
|  | 23.55 |  |  | 22.6 | -1.066666667 |  | -7.09 | 136.2393834 |  |  |  |  |
| 20 (+LPS) | 23.02 | 23.1666667 | 0.1305 | 22.07 | -1.096666667 | -1.13 | -7.12 | 139.1020624 | 142.45538 | 6.658 | 0.000002 | ** |
|  | 23.21 |  |  | 22.08 | -1.086666667 |  | -7.11 | 138.1412143 |  |  |  |  |
|  | 23.27 |  |  | 21.96 | -1.206666667 |  | -7.23 | 150.1228735 |  |  |  |  |
| 40 (+LPS) | 23.52 | 23.5433333 | 0.0586 | 23.23 | -0.313333333 | -0.48 | -6.33666667 | 80.82146888 | 91.140855 | 10.76 | 0.000002 | ** |
|  | 23.5 |  |  | 23.07 | -0.473333333 |  | -6.49666667 | 90.30078766 |  |  |  |  |
|  | 23.61 |  |  | 22.89 | -0.653333333 |  | -6.67666667 | 102.3003072 |  |  |  |  |
| 80 (+LPS) | 24.12 | 24.0133333 | 0.0924 | 23.9 | -0.113333333 | -0.07 | -6.13666667 | 70.35917526 | 68.839164 | 10.61 | 0.000001 | ** |
|  | 23.96 |  |  | 23.74 | -0.273333333 |  | -6.29666667 | 78.61140156 |  |  |  |  |
|  | 23.96 |  |  | 24.19 | 0.176666667 |  | -5.84666667 | 57.54691428 |  |  |  |  |
|  |  | | | | | | | | | |  |  |
|  |  |  |  |  |  |  |  |  |  |  |  |  |
|  |  |  |  |  |  |  |  |  |  |  |  |  |
|  |  |  |  |  |  |  |  |  |  |  |  |  |
|  |  |  |  |  |  |  |  |  |  |  |  |  |
|  |  |  |  |  |  |  |  |  |  |  |  |  |
|  |  |  |  |  |  |  |  |  |  |  |  |  |
|  |  |  |  |  |  |  |  |  |  |  |  |  |
|  |  |  |  |  |  |  |  |  |  |  |  |  |
|  |  |  |  |  |  |  |  |  |  |  |  |  |
|  |  |  |  |  |  |  |  |  |  |  |  |  |
|  |  | | | | | | | | | |  |  |

| **sample** | **β-actin** | **average** | **β-actin Std.** | **COX-2** | **COX-2 /β-actin** | **average (ΔCt)** | **ΔΔCt** | **2^-(ΔΔCt)** | **ΔΔCt average** | **ΔΔCt STD** | **t-test** | **p value** |
| --- | --- | --- | --- | --- | --- | --- | --- | --- | --- | --- | --- | --- |
| - (control) | 23.42 | 23.4766667 | 0.0981495 | 19.16 | -4.316666667 | -4.38 | 0.06333333 | 0.957050307 | 1.004415 | 0.1174 |  |  |
|  | 23.59 |  |  | 18.91 | -4.566666667 |  | -0.18666667 | 1.138131035 |  |  |  |  |
|  | 23.42 |  |  | 19.22 | -4.256666667 |  | 0.12333333 | 0.91806402 |  |  |  |  |
| + (LPS) | 23.36 | 23.4833333 | 0.2136196 | 13.3 | -10.18333333 | -10.116667 | -5.80333333 | 55.84411441 | 53.40439 | 3.5806 | 0.000007 | # |
|  | 23.36 |  |  | 13.32 | -10.16333333 |  | -5.78333333 | 55.07529198 |  |  |  |  |
|  | 23.73 |  |  | 13.48 | -10.00333333 |  | -5.62333333 | 49.29376711 |  |  |  |  |
| 10 (+LPS) | 23.48 | 23.6666667 | 0.2650157 | 13.99 | -9.676666667 | -9.73 | -5.29666667 | 39.30570078 | 40.81643 | 1.9457 | 0.002942 | ** |
|  | 23.97 |  |  | 13.86 | -9.806666667 |  | -5.42666667 | 43.01198079 |  |  |  |  |
|  | 23.55 |  |  | 13.96 | -9.706666667 |  | -5.32666667 | 40.13159711 |  |  |  |  |
| 20 (+LPS) | 23.02 | 23.1666667 | 0.1305118 | 14.44 | -8.726666667 | -8.9166667 | -4.34666667 | 20.34590666 | 23.32042 | 2.744 | 0.000160 | ** |
|  | 23.21 |  |  | 14.21 | -8.956666667 |  | -4.57666667 | 23.86239041 |  |  |  |  |
|  | 23.27 |  |  | 14.1 | -9.066666667 |  | -4.68666667 | 25.75296552 |  |  |  |  |
| 40 (+LPS) | 23.52 | 23.5433333 | 0.0585947 | 14.6 | -8.943333333 | -8.8433333 | -4.56333333 | 23.64287105 | 22.09492 | 1.5306 | 0.000077 | ** |
|  | 23.5 |  |  | 14.7 | -8.843333333 |  | -4.46333333 | 22.0595787 |  |  |  |  |
|  | 23.61 |  |  | 14.8 | -8.743333333 |  | -4.36333333 | 20.58231471 |  |  |  |  |
| 80 (+LPS) | 24.12 | 24.0133333 | 0.092376 | 15.7 | -8.313333333 | -8.24 | -3.93333333 | 15.27746566 | 14.72107 | 2.905 | 0.000065 | ** |
|  | 23.96 |  |  | 15.52 | -8.493333333 |  | -4.11333333 | 17.30759466 |  |  |  |  |
|  | 23.96 |  |  | 16.1 | -7.913333333 |  | -3.53333333 | 11.5781539 |  |  |  |  |
|  | \|  \| \| --- \| |  |  |  |  |  |  |  |  |  |  |  |
|  |  |  |  |  |  |  |  |  |  |  |  |  |
|  |  |  |  |  |  |  |  |  |  |  |  |  |
|  |  |  |  |  |  |  |  |  |  |  |  |  |
|  |  |  |  |  |  |  |  |  |  |  |  |  |
|  |  |  |  |  |  |  |  |  |  |  |  |  |
|  |  |  |  |  |  |  |  |  |  |  |  |  |
|  |  |  |  |  |  |  |  |  |  |  |  |  |
|  |  |  |  |  |  |  |  |  |  |  |  |  |
|  |  |  |  |  |  |  |  |  |  |  |  |  |
|  |  |  |  |  |  |  |  |  |  |  |  |  |

**Table S2: Results of the COX-2 expression in LPS-stimulated RAW 264.7 cells.**

**Table S3: Results of the IL-6 expression in LPS-stimulated RAW 264.7 cells.**

| **sample** | **β-actin** | **average** | **β-actin Std.** | **IL-6** | **IL-6/β-actin** | **average (ΔCt)** | **ΔΔCt** | **2^-(ΔΔCt)** | **ΔΔCt average** | **ΔΔCt STD** | **t-test** | **p value** |
| --- | --- | --- | --- | --- | --- | --- | --- | --- | --- | --- | --- | --- |
| - (control) | 23.42 | 23.47666667 | 0.09815 | 31.69 | 8.213333333 | 8.446667 | -0.233333333 | 1.175547906 | 1.015982 | 0.21189 |  |  |
|  | 23.59 |  |  | 32.29 | 8.813333333 |  | 0.366666667 | 0.775572381 |  |  |  |  |
|  | 23.42 |  |  | 31.79 | 8.313333333 |  | -0.133333333 | 1.09682498 |  |  |  |  |
| + (LPS) | 23.36 | 23.48333333 | 0.21362 | 21.04 | -2.443333333 | -2.52333 | -10.89 | 1897.652351 | 2008.57 | 128.791 | 0.000006 | # |
|  | 23.36 |  |  | 20.86 | -2.623333333 |  | -11.07 | 2149.819768 |  |  |  |  |
|  | 23.73 |  |  | 20.98 | -2.503333333 |  | -10.95 | 1978.237602 |  |  |  |  |
| 10 (+LPS) | 23.48 | 23.66666667 | 0.265016 | 21.75 | -1.916666667 | -1.8 | -10.36333333 | 1317.268141 | 1218.302 | 109.895 | 0.000636 | ** |
|  | 23.97 |  |  | 21.84 | -1.826666667 |  | -10.27333333 | 1237.603386 |  |  |  |  |
|  | 23.55 |  |  | 22.01 | -1.656666667 |  | -10.10333333 | 1100.034712 |  |  |  |  |
| 20 (+LPS) | 23.02 | 23.16666667 | 0.130512 | 22.5 | -0.666666667 | -0.61667 | -9.113333333 | 553.843029 | 535.3092 | 22.9495 | 0.000020 | ** |
|  | 23.21 |  |  | 22.53 | -0.636666667 |  | -9.083333333 | 542.4451043 |  |  |  |  |
|  | 23.27 |  |  | 22.62 | -0.546666667 |  | -8.993333333 | 509.6395157 |  |  |  |  |
| 40 (+LPS) | 23.52 | 23.54333333 | 0.058595 | 24.24 | 0.696666667 | 0.626667 | -7.75 | 215.2694823 | 226.4387 | 18.0644 | 0.000009 | ** |
|  | 23.5 |  |  | 24.23 | 0.686666667 |  | -7.76 | 216.7668 |  |  |  |  |
|  | 23.61 |  |  | 24.04 | 0.496666667 |  | -7.95 | 247.2797002 |  |  |  |  |
| 80 (+LPS) | 24.12 | 24.01333333 | 0.092376 | 25.89 | 1.876666667 | 2.293333 | -6.57 | 95.00950852 | 73.11627 | 20.7183 | 0.000007 | ** |
|  | 23.96 |  |  | 26.32 | 2.306666667 |  | -6.14 | 70.52192742 |  |  |  |  |
|  | 23.96 |  |  | 26.71 | 2.696666667 |  | -5.75 | 53.81737058 |  |  |  |  |
|  |  |  | | | | | | | | |  |  |
|  |  |  |  |  |  |  |  |  |  |  |  |  |
|  |  |  |  |  |  |  |  |  |  |  |  |  |
|  |  |  |  |  |  |  |  |  |  |  |  |  |
|  |  |  |  |  |  |  |  |  |  |  |  |  |
|  |  |  |  |  |  |  |  |  |  |  |  |  |
|  |  |  |  |  |  |  |  |  |  |  |  |  |
|  |  |  |  |  |  |  |  |  |  |  |  |  |
|  |  |  |  |  |  |  |  |  |  |  |  |  |
|  |  |  |  |  |  |  |  |  |  |  |  |  |
|  |  |  |  |  |  |  |  |  |  |  |  |  |
|  |  |  |  |  |  |  |  |  |  |  |  |  |

**Table S4: Results of the TNF-α expression in LPS-stimulated RAW 264.7 cells.**

| **sample** | **β-actin** | **average** | **β-actin Std.** | **TNF-α** | **TNF-α/β-actin (ΔCt)** | **average (ΔCt)** | **ΔΔCt** | **2^-(ΔΔCt)** | **ΔΔCt average** | **ΔΔCt STD** | **t-test** | **p value** |
| --- | --- | --- | --- | --- | --- | --- | --- | --- | --- | --- | --- | --- |
| - (control) | 22.16 | 22.22666667 | 0.0702 | 18.48 | -3.7466666667 | -3.74 | -0.006666667 | 1.004631674 | 1.00002 | 0.007985 |  |  |
|  | 22.3 |  |  | 18.5 | -3.7266666667 |  | 0.013333333 | 0.990800613 |  |  |  |  |
|  | 22.22 |  |  | 18.48 | -3.7466666667 |  | -0.006666667 | 1.004631674 |  |  |  |  |
| + (LPS) | 22.15 | 22.36 | 0.1852 | 13.92 | -8.4400000000 | -8.376666667 | -4.7 | 25.99207668 | 25.0214 | 3.24013 | 0.000106 | # |
|  | 22.5 |  |  | 14.2 | -8.1600000000 |  | -4.42 | 21.40684088 |  |  |  |  |
|  | 22.43 |  |  | 13.83 | -8.5300000000 |  | -4.79 | 27.6651914 |  |  |  |  |
| 10 (+LPS) | 22.15 | 22.24333333 | 0.1137 | 14.34 | -7.9033333333 | -7.72 | -4.163333333 | 17.91794566 | 15.871 | 2.075113 | 0.007312 | ** |
|  | 22.21 |  |  | 14.51 | -7.7333333333 |  | -3.993333333 | 15.92623487 |  |  |  |  |
|  | 22.37 |  |  | 14.72 | -7.5233333333 |  | -3.783333333 | 13.768823 |  |  |  |  |
| 20 (+LPS) | 21.99 | 21.94666667 | 0.1115 | 14.73 | -7.2166666667 | -7.283333333 | -3.476666667 | 11.13219875 | 11.684 | 0.955711 | 0.001196 | ** |
|  | 22.03 |  |  | 14.73 | -7.2166666667 |  | -3.476666667 | 11.13219875 |  |  |  |  |
|  | 21.82 |  |  | 14.53 | -7.4166666667 |  | -3.676666667 | 12.7875384 |  |  |  |  |
| 40 (+LPS) | 22.37 | 22.31666667 | 0.215 | 15.11 | -7.2066666667 | -7.226666667 | -3.466666667 | 11.05530304 | 11.2362 | 0.952927 | 0.001056 | ** |
|  | 22.5 |  |  | 15.2 | -7.1166666667 |  | -3.376666667 | 10.38670871 |  |  |  |  |
|  | 22.08 |  |  | 14.96 | -7.3566666667 |  | -3.616666667 | 12.26662676 |  |  |  |  |
| 80 (+LPS) | 22.52 | 22.69333333 | 0.3265 | 15.51 | -7.1833333333 | -7.076666667 | -3.443333333 | 10.87793898 | 10.1223 | 0.769619 | 0.000747 | ** |
|  | 23.07 |  |  | 15.61 | -7.0833333333 |  | -3.343333333 | 10.14947595 |  |  |  |  |
|  | 22.49 |  |  | 15.73 | -6.9633333333 |  | -3.223333333 | 9.33942243 |  |  |  |  |
|  |  | | | | | | | | |  |  |  |
|  |  |  |  |  |  |  |  |  |  |  |  |  |
|  |  |  |  |  |  |  |  |  |  |  |  |  |
|  |  |  |  |  |  |  |  |  |  |  |  |  |
|  |  |  |  |  |  |  |  |  |  |  |  |  |
|  |  |  |  |  |  |  |  |  |  |  |  |  |
|  |  |  |  |  |  |  |  |  |  |  |  |  |
|  |  |  |  |  |  |  |  |  |  |  |  |  |
|  |  |  |  |  |  |  |  |  |  |  |  |  |
|  |  |  |  |  |  |  |  |  |  |  |  |  |
|  |  |  |  |  |  |  |  |  |  |  |  |  |

**Table S5: Results of the iNOS expression in zebrafish larvae.**

| **sample** | **β-actin** | **average** | **β-actin Std.** | **iNOS** | **iNOS/β-actin** | **average (ΔCt)** | **ΔΔCt** | **2^-(ΔΔCt)** | **ΔΔCt average** | **ΔΔCt STD** | **t-test** | **p value** |
| --- | --- | --- | --- | --- | --- | --- | --- | --- | --- | --- | --- | --- |
| 0 (control) | 18.08 | 17.85333333 | 0.537153 | 24.99 | 7.13667 | 6.760000000 | 0.376666667 | 0.770215111 | 1.031239936 | 0.3200475 |  |  |
|  | 18.24 |  |  | 24.71 | 6.85667 |  | 0.096666667 | 0.935191248 |  |  |  |  |
|  | 17.24 |  |  | 24.14 | 6.28667 |  | -0.473333333 | 1.38831345 |  |  |  |  |
| 0 (tail section) | 18 | 17.83333333 | 0.2081666 | 22.99 | 5.15667 | 4.970000000 | -1.603333333 | 3.038445328 | 3.47522064 | 0.4185059 | 0.000651 | # |
|  | 17.9 |  |  | 22.78 | 4.94667 |  | -1.813333333 | 3.514533809 |  |  |  |  |
|  | 17.6 |  |  | 22.64 | 4.80667 |  | -1.953333333 | 3.872682784 |  |  |  |  |
| DEX | 18 | 18.18333333 | 1.2947715 | 23.1 | 4.91667 | 5.926666667 | -1.843333333 | 3.588381635 | 1.300535525 | 0.4794926 | 0.247434 |  |
|  | 19.56 |  |  | 24.23 | 6.04667 |  | -0.713333333 | 1.639587997 |  |  |  |  |
|  | 16.99 |  |  | 25 | 6.81667 |  | 0.056666667 | 0.961483052 |  |  |  |  |
| 25ug/ml | 19.4 | 19.26666667 | 0.2055075 | 24.44 | 5.17333 | 5.023333333 | -1.586666667 | 3.003545807 | 3.356822029 | 0.5054719 | 0.001268 | ** |
|  | 19.37 |  |  | 24.38 | 5.11333 |  | -1.64667 | 3.131093665 |  |  |  |  |
|  | 19.03 |  |  | 24.05 | 4.78333 |  | -1.976666667 | 3.935826614 |  |  |  |  |
| 50ug/ml | 20.39 | 18.87333333 | 1.3194064 | 25.48 | 6.60667 | 5.586666667 | -0.153333333 | 1.112136086 | 3.213620554 | 0.1574459 | 0.001630 | ** |
|  | 17.99 |  |  | 23.9 | 5.02667 |  | -1.733333333 | 3.324951585 |  |  |  |  |
|  | 18.24 |  |  | 24 | 5.12667 |  | -1.633333333 | 3.102289524 |  |  |  |  |
| 100ug/ml | 18.79 | 18.76 | 0.1868154 | 23.97 | 5.21000 | 5.686666667 | -1.550000000 | 2.928171392 | 1.785667883 | 0.1137001 | 0.027340 | * |
|  | 18.56 |  |  | 24.62 | 5.86000 |  | -0.900000000 | 1.866065983 |  |  |  |  |
|  | 18.93 |  |  | 24.75 | 5.99000 |  | -0.770000000 | 1.705269784 |  |  |  |  |
|  | \|  \| \| --- \| |  |  |  |  |  |  |  |  |  |  |  |
|  |  |  |  |  |  |  |  |  |  |  |  |  |
|  |  |  |  |  |  |  |  |  |  |  |  |  |
|  |  |  |  |  |  |  |  |  |  |  |  |  |
|  |  |  |  |  |  |  |  |  |  |  |  |  |
|  |  |  |  |  |  |  |  |  |  |  |  |  |
|  |  |  |  |  |  |  |  |  |  |  |  |  |
|  |  |  |  |  |  |  |  |  |  |  |  |  |
|  |  |  |  |  |  |  |  |  |  |  |  |  |
|  |  |  |  |  |  |  |  |  |  |  |  |  |
|  |  |  |  |  |  |  |  |  |  |  |  |  |
|  |  |  |  |  |  |  |  |  |  |  |  |  |

**Table S6: Results of the COX-2 expression in zebrafish larvae.**

| **sample** | **β-actin** | **average** | **β-actin Std.** | **COX-2** | COX-2/β-actin | **average (ΔCt)** | **ΔΔCt** | **2^-(ΔΔCt)** | **ΔΔCt average** | **ΔΔCt STD** | **t-test** | **p value** |
| --- | --- | --- | --- | --- | --- | --- | --- | --- | --- | --- | --- | --- |
| 0 (control) | 16.48 | 15.95333333 | 1.035825 | 21.36 | 5.40666667 | 4.3333333 | 1.0733 | 0.475219739 | 1.454835616 | 0.156569304 |  |  |
|  | 16.62 |  |  | 19.64 | 3.68666667 |  | -0.6467 | 1.565546833 |  |  |  |  |
|  | 14.76 |  |  | 19.86 | 3.90666667 |  | -0.4267 | 1.344124400 |  |  |  |  |
| 0 (tail section) | 15.96 | 16.40333333 | 1.283758 | 18.4 | 1.99666667 | 2.7533333 | -2.3367 | 5.051341805 | 2.300729187 | 0.078920316 | 0.010407 | # |
|  | 15.4 |  |  | 19.57 | 3.16666667 |  | -1.1667 | 2.244924097 |  |  |  |  |
|  | 17.85 |  |  | 19.5 | 3.09666667 |  | -1.2367 | 2.356534278 |  |  |  |  |
| DEX | 16.78 | 16.41666667 | 1.255083 | 20.7 | 4.28333333 | 4.0600000 | -0.0500 | 1.035264924 | 1.035264924 | 0 | 0.031553 | * |
|  | 15.02 |  |  | 20.7 | 4.28333333 |  | -0.0500 | 1.035264924 |  |  |  |  |
|  | 17.45 |  |  | 20.03 | 3.61333333 |  | -0.7200 | 1.647182035 |  |  |  |  |
| 25ug/ml | 19.61 | 17.92333333 | 1.48544 | 21.42 | 3.49666667 | 3.1633333 | -0.8367 | 1.785919022 | 2.285927429 | 0.48932815 | 0.056389 | * |
|  | 17.35 |  |  | 21.05 | 3.12666667 |  | -1.2067 | 2.308037504 |  |  |  |  |
|  | 16.81 |  |  | 20.79 | 2.86666667 |  | -1.4667 | 2.763825760 |  |  |  |  |
| 50ug/ml | 17.06 | 16.39333333 | 0.869387 | 21.53 | 5.13666667 | 4.1766667 | 0.8033 | 0.573023681 | 1.564303908 | 0.244346496 | 0.323532 |  |
|  | 16.71 |  |  | 20.25 | 3.85666667 |  | -0.4767 | 1.391524844 |  |  |  |  |
|  | 15.41 |  |  | 19.93 | 3.53666667 |  | -0.7967 | 1.737082973 |  |  |  |  |
| 100ug/ml | 16.44 | 14.45 | 2.052632 | 20.93 | 6.48000000 | 5.4033333 | 2.1467 | 0.225833800 | 0.261911643 | 0.051021775 | 0.004697 | ** |
|  | 12.34 |  |  | 20.53 | 6.08000000 |  | 1.7467 | 0.297989486 |  |  |  |  |
|  | 14.57 |  |  | 18.1 | 3.65000000 |  | -0.6833 | 1.605845764 |  |  |  |  |
|  | \|  \| \| --- \| |  |  |  |  |  |  |  |  |  |  |  |
|  |  |  |  |  |  |  |  |  |  |  |  |  |
|  |  |  |  |  |  |  |  |  |  |  |  |  |
|  |  |  |  |  |  |  |  |  |  |  |  |  |
|  |  |  |  |  |  |  |  |  |  |  |  |  |
|  |  |  |  |  |  |  |  |  |  |  |  |  |
|  |  |  |  |  |  |  |  |  |  |  |  |  |
|  |  |  |  |  |  |  |  |  |  |  |  |  |
|  |  |  |  |  |  |  |  |  |  |  |  |  |
|  |  |  |  |  |  |  |  |  |  |  |  |  |
|  |  |  |  |  |  |  |  |  |  |  |  |  |
|  |  |  |  |  |  |  |  |  |  |  |  |  |

**Table S7: Results of the TNF-α expression in zebrafish larvae.**

| **sample** | **β-actin** | **average** | **β-actin Std.** | **TNF-α** | **TNF-a/β-actin** | **average (ΔCt)** | **ΔΔCt** | **2^-(ΔΔCt)** | **ΔΔCt average** | **ΔΔCt STD** | **t-test** | **p value** |
| --- | --- | --- | --- | --- | --- | --- | --- | --- | --- | --- | --- | --- |
| 0 (control) | 16.48 | 15.95333333 | 1.03582 | 19.1 | 3.14666667 | 5.2266667 | -2.08 | 4.22807216 | 1.00856 | 1.249539 |  |  |
|  | 16.62 |  |  | 24.18 | 8.22666667 |  | 3 | 0.12500000 |  |  |  |  |
|  | 14.76 |  |  | 20.26 | 4.30666667 |  | -0.92 | 1.89211529 |  |  |  |  |
| 0 (tail section) | 15.96 | 16.40333333 | 1.28376 | 19.1 | 2.69666667 | 2.3033333 | -2.53 | 5.77571678 | 8.71916 | 0.938356 | 0.009962 | # |
|  | 15.4 |  |  | 18.62 | 2.21666667 |  | -3.01 | 8.05564440 |  |  |  |  |
|  | 17.85 |  |  | 18.4 | 1.99666667 |  | -3.23 | 9.38267959 |  |  |  |  |
| DEX | 16.78 | 16.41666667 | 1.25508 | 20.51 | 4.09333333 | 4.19 | -1.133333333 | 2.19364996 | 2.12615 | 0.667616 | 0.134358 |  |
|  | 15.02 |  |  | 21.13 | 4.71333333 |  | -0.513333333 | 1.42734425 |  |  |  |  |
|  | 17.45 |  |  | 20.18 | 3.76333333 |  | -1.463333333 | 2.75744734 |  |  |  |  |
| 25ug/ml | 17.35 | 18.25666667 | 1.19438 | 22.36 | 4.10333333 | 3.34 | -1.123333333 | 2.17849731 | 4.84921 | 0.780921 | 0.033178 | * |
|  | 19.61 |  |  | 21.38 | 3.12333333 |  | -2.103333333 | 4.29701059 |  |  |  |  |
|  | 17.81 |  |  | 21.05 | 2.79333333 |  | -2.433333333 | 5.40139978 |  |  |  |  |
| 50ug/ml | 17.06 | 16.39333333 | 0.86939 | 23.5 | 7.10666667 | 5.2133333 | 1.88 | 0.27168372 | 1.98206 | 0.537292 | 0.208992 |  |
|  | 16.71 |  |  | 20.94 | 4.54666667 |  | -0.68 | 1.60213976 |  |  |  |  |
|  | 15.41 |  |  | 20.38 | 3.98666667 |  | -1.24 | 2.36198532 |  |  |  |  |
| 100ug/ml | 16.44 | 14.45 | 2.05263 | 19.09 | 4.64000000 | 5.1166667 | -0.586666667 | 1.50177290 | 1.73484 | 0.329611 | 0.255029 |  |
|  | 12.34 |  |  | 18.7 | 4.25000000 |  | -0.976666667 | 1.96791331 |  |  |  |  |
|  | 14.57 |  |  | 20.91 | 6.46000000 |  | 1.233333333 | 0.42533358 |  |  |  |  |
|  | \|  \| \| --- \| |  |  |  |  |  |  |  |  |  |  |  |
|  |  |  |  |  |  |  |  |  |  |  |  |  |
|  |  |  |  |  |  |  |  |  |  |  |  |  |
|  |  |  |  |  |  |  |  |  |  |  |  |  |
|  |  |  |  |  |  |  |  |  |  |  |  |  |
|  |  |  |  |  |  |  |  |  |  |  |  |  |
|  |  |  |  |  |  |  |  |  |  |  |  |  |
|  |  |  |  |  |  |  |  |  |  |  |  |  |
|  |  |  |  |  |  |  |  |  |  |  |  |  |
|  |  |  |  |  |  |  |  |  |  |  |  |  |
|  |  |  |  |  |  |  |  |  |  |  |  |  |
|  |  |  |  |  |  |  |  |  |  |  |  |  |

**Table S8: Results of the IL-10 expression in zebrafish larvae.**

| **sample** | **β-actin** | **average** | **β-actin Std.** | **IL-10** | **IL-10/β-actin** | **average (ΔCt)** | **ΔΔCt** | **2^-(ΔΔCt)** | **ΔΔCt average** | **ΔΔCt STD** | **t-test** | **p value** |
| --- | --- | --- | --- | --- | --- | --- | --- | --- | --- | --- | --- | --- |
| 0 (control) | 17.16 | 17.48333333 | 1.43263 | 23.16 | 5.676666667 | 6.3 | -0.623333333 | 1.540430222 | 0.8061024 | 0.03555 |  |  |
|  | 19.05 |  |  | 24.14 | 6.656666667 |  | 0.356666667 | 0.780966913 |  |  |  |  |
|  | 16.24 |  |  | 24.05 | 6.566666667 |  | 0.266666667 | 0.831237896 |  |  |  |  |
| 0 (tail section) | 16.33 | 17.57666667 | 1.08339 | 22.51 | 4.933333333 | 4.73 | -1.366666667 | 2.578740617 | 2.294002 | 0.40268 | 0.017491 | # |
|  | 18.29 |  |  | 22.87 | 5.293333333 |  | -1.006666667 | 2.009263349 |  |  |  |  |
|  | 18.11 |  |  | 21.54 | 3.963333333 |  | -2.336666667 | 5.051341805 |  |  |  |  |
| DEX | 14.97 | 15.23666667 | 0.33307 | 20.44 | 5.203333333 | 7.95 | -1.096666667 | 2.138599997 | 0.1230212 | 0.00362 | 0.000683 | ** |
|  | 15.61 |  |  | 24.53 | 9.293333333 |  | 2.993333333 | 0.125578959 |  |  |  |  |
|  | 15.13 |  |  | 24.59 | 9.353333333 |  | 3.053333333 | 0.12046339 |  |  |  |  |
| 25ug/ml | 19.53 | 18.6 | 0.80542 | 25.91 | 7.310000000 | 6.03 | 1.01 | 0.496546248 | 1.9009355 | 0.4068 | 0.031525 | * |
|  | 18.13 |  | 0.15885 | 24.21 | 5.610000000 |  | -0.69 | 1.613283518 |  |  |  |  |
|  | 18.14 |  | 0.13577 | 23.77 | 5.170000000 |  | -1.13 | 2.188587403 |  |  |  |  |
| 50ug/ml | 18.41 | 18.18333333 | 0.30238 | 23.94 | 5.756666667 | 5.283333 | -0.543333333 | 1.457335791 | 1.6638591 | 0.29207 | 0.027050 | * |
|  | 18.3 |  |  | 23.58 | 5.396666667 |  | -0.903333333 | 1.870382496 |  |  |  |  |
|  | 17.84 |  |  | 22.88 | 4.696666667 |  | -1.603333333 | 3.038445328 |  |  |  |  |
| 100ug/ml | 19.54 | 18.55666667 | 0.85172 | 26.48 | 7.923333333 | 6.623333 | 1.623333333 | 0.324584647 | 1.256041 | 0.0984 | 0.012993 | * |
|  | 18.08 |  |  | 24.61 | 6.053333333 |  | -0.246666667 | 1.186462635 |  |  |  |  |
|  | 18.05 |  |  | 24.45 | 5.893333333 |  | -0.406666667 | 1.325619442 |  |  |  |  |
|  |  |  | | | | | | | |  |  |  |
|  |  |  |  |  |  |  |  |  |  |  |  |  |
|  |  |  |  |  |  |  |  |  |  |  |  |  |
|  |  |  |  |  |  |  |  |  |  |  |  |  |
|  |  |  |  |  |  |  |  |  |  |  |  |  |
|  |  |  |  |  |  |  |  |  |  |  |  |  |
|  |  |  |  |  |  |  |  |  |  |  |  |  |
|  |  |  |  |  |  |  |  |  |  |  |  |  |
|  |  |  |  |  |  |  |  |  |  |  |  |  |
|  |  |  |  |  |  |  |  |  |  |  |  |  |
|  |  |  |  |  |  |  |  |  |  |  |  |  |
|  |  |  |  |  |  |  |  |  |  |  |  |  |

**Table S9: Results of the IL-1β expression in zebrafish larvae.**

| **sample** | **β-actin** | **average** | **β-actin Std.** | **IL-1b** | **IL-1b/β-actin** | **average (ΔCt)** | **ΔΔCt** | **2^-(ΔΔCt)** | **ΔΔCt average** | **ΔΔCt STD** | **t-test** | **p value** |
| --- | --- | --- | --- | --- | --- | --- | --- | --- | --- | --- | --- | --- |
| 0 (control) | 17.89 | 17.82 | 0.767398202 | 22.8 | 4.980000000 | 5.193333 | -0.213333333 | 1.159363791 | 1.023919 | 0.255985 |  |  |
|  | 18.55 |  |  | 23.47 | 5.650000000 |  | 0.456666667 | 0.728667896 |  |  |  |  |
|  | 17.02 |  |  | 22.77 | 4.950000000 |  | -0.243333333 | 1.183724489 |  |  |  |  |
| 0 (tail section) | 17.57 | 17.81333333 | 0.220529665 | 20.98 | 3.166666667 | ####### | -2.026666667 | 4.07462324 | 2.337239 | 0.444063 | 0.011224 | # |
|  | 17.87 |  |  | 21.6 | 3.786666667 |  | -1.406666667 | 2.651238884 |  |  |  |  |
|  | 18 |  |  | 21.99 | 4.176666667 |  | -1.016666667 | 2.023238881 |  |  |  |  |
| DEX | 17.68 | 18.95333333 | 1.153964182 | 22.48 | 3.526666667 | 4.243333 | -1.666666667 | 3.174802104 | 1.508082 | 0.081268 | 0.044796 | * |
|  | 19.93 |  |  | 23.5 | 4.546666667 |  | -0.646666667 | 1.565546833 |  |  |  |  |
|  | 19.25 |  |  | 23.61 | 4.656666667 |  | -0.536666667 | 1.450617005 |  |  |  |  |
| 25ug/ml | 18.41 | 19.43 | 1.663850955 | 22.95 | 3.520000000 | ####### | -1.673333333 | 3.189506754 | 1.904503 | 0.796883 | 0.076144 |  |
|  | 21.35 |  |  | 23.32 | 3.890000000 |  | -1.303333333 | 2.467984499 |  |  |  |  |
|  | 18.53 |  |  | 24.2 | 4.770000000 |  | -0.423333333 | 1.341022398 |  |  |  |  |
| 50ug/ml | 19.08 | 18.75666667 | 0.335608899 | 24.89 | 6.133333333 | 5.013333 | 0.94 | 0.52123288 | 0.830998 | 0.438074 | 0.282685 |  |
|  | 18.41 |  |  | 23.76 | 5.003333333 |  | -0.19 | 1.140763716 |  |  |  |  |
|  | 18.78 |  |  | 22.66 | 3.903333333 |  | -1.29 | 2.445280555 |  |  |  |  |
| 100ug/ml | 18.23 | 17.06666667 | 1.525133874 | 25.31 | 8.243333333 | ####### | 3.05 | 0.120742041 | 0.124399 | 0.116979 | 0.002602 | ** |
|  | 17.63 |  |  | 24.3 | 7.233333333 |  | 2.04 | 0.243163737 |  |  |  |  |
|  | 15.34 |  |  | 29.01 | 11.94333333 |  | 6.75 | 0.009290681 |  |  |  |  |
|  |  | | | | | | | |  |  |  |  |
|  |  |  |  |  |  |  |  |  |  |  |  |  |
|  |  |  |  |  |  |  |  |  |  |  |  |  |
|  |  |  |  |  |  |  |  |  |  |  |  |  |
|  |  |  |  |  |  |  |  |  |  |  |  |  |
|  |  |  |  |  |  |  |  |  |  |  |  |  |
|  |  |  |  |  |  |  |  |  |  |  |  |  |
|  |  |  |  |  |  |  |  |  |  |  |  |  |
|  |  |  |  |  |  |  |  |  |  |  |  |  |
|  |  |  |  |  |  |  |  |  |  |  |  |  |
|  |  |  |  |  |  |  |  |  |  |  |  |  |
|  |  |  |  |  |  |  |  |  |  |  |  |  |
